# A one-year study of foodborne illnesses in the municipality of Uppsala, Sweden.

**DOI:** 10.3201/eid0707.010742

**Published:** 2001

**Authors:** R Lindqvist, Y Andersson, J Lindbäck, M Wegscheider, Y Eriksson, L Tideström, A Lagerqvist-Widh, K O Hedlund, S Löfdahl, L Svensson, A Norinder

**Affiliations:** National Food Administration, Uppsala, Sweden. roli@slv.se

## Abstract

Surveillance was enhanced and a retrospective interview study performed in 1998-99 to determine incidence, causes, and costs of foodborne illnesses in Uppsala, Sweden. Sixty-eight percent of the detected foodborne illness incidents were single cases, and 32% were outbreaks. Most (85%) of the incidents came to the attention of the municipal authorities through telephone calls from affected persons. Calicivirus, Campylobacter spp., and Staphyloccocus aureus were the most common etiological agents; meat, meat products, and mixed dishes were the most implicated food categories. The incidence of foodborne illness was estimated to be 38 cases per 1,000 inhabitants per year. The estimated average costs per illness were 2,164 Swedish Krona (SEK) ($246) to society and 500 SEK ($57) to the patient. The annual cost of foodborne illnesses in Sweden was estimated to be 1,082 million SEK ($123 million).


### A One-Year Study of Foodborne Illnesses in the Municipality of Uppsala, Sweden

**Roland Lindqvist,* Yvonne Andersson,† Johan Lindbäck,† Maria Wegscheider,‡ Yvonne Eriksson,‡ Lasse Tideström,§ Angela Lagerqvist-Widh,¶ Kjell-Olof Hedlund,† Sven Löfdahl,† Lennart Svensson,† and Anna Norinder#**

*National Food Administration, Uppsala, Sweden; †Swedish Institute for Infectious Diseases Control, Solna, Sweden; ‡The Municipality of Uppsala, Uppsala, Sweden; §The Regional Infectious Disease Unit, Uppsala, Sweden; ¶University Hospital, Uppsala, Sweden; and #The Swedish Institute for Health Economics, Lund, Sweden

Surveillance was enhanced and a retrospective interview study performed in 1998–99 to determine incidence, causes, and costs of foodborne illnesses in Uppsala, Sweden. Sixty-eight percent of the detected foodborne illness incidents were single cases, and 32% were outbreaks. Most (85%) of the incidents came to the attention of the municipal authorities through telephone calls from affected persons. Calicivirus, *Campylobacter* spp., and *Staphylococcus aureus* were the most common etiological agents; meat, meat products, and mixed dishes were the most implicated food categories. The incidence of foodborne illness was estimated to be 38 cases per 1,000 inhabitants per year. The estimated average costs per illness were 2,164 Swedish Krona (SEK) ($246) to society and 500 SEK ($57) to the patient. The annual cost of foodborne illnesses in Sweden was estimated to be 1,082 million SEK ($123 million).

Foodborne illnesses are a widespread global problem (1). In most cases, the clinical picture is mild and self-limiting, with few deaths. However, the socioeconomic impact may be high (2-4). Possible chronic sequelae, which have been estimated to occur in 2% to 3% of cases (5,6), may add to the suffering and costs associated with foodborne illnesses. For several reasons, foodborne illnesses are seriously underreported (7), but investigation and surveillance remain essential in efforts to understand and prevent them (8,9).

In Sweden, 794 to 2,965 cases of foodborne illness were reported yearly from 1992 to 1997 (10). In contrast, findings from a 1994 interview study in Sweden indicated that 500,000 persons per year experienced foodborne illnesses (11). This discrepancy illustrates both our lack of knowledge of the true extent of the problem as well as difficulties in reporting.

The aim of this study was to improve our understanding of foodborne illnesses. The specific objectives were 1) to detect and investigate as many outbreaks and single cases as possible in the municipality of Uppsala and determine the specific causes behind these illnesses and 2) to estimate the incidence and costs associated with foodborne illnesses.

### Material and Methods

### Surveillance of Foodborne Illnesses in Sweden

In Sweden, the municipal public health authorities are responsible for preventing the spread of foodborne illnesses, whereas the County Medical Officer (CMO) at the County Council has coordinating responsibility for communicable diseases and other foodborne diseases. Physicians are responsible for epidemiologic investigations in relation to their patients and should report communicable diseases to the CMO and the Swedish Institute for Infectious Disease Control. In addition, physicians should report communicable diseases that may be contracted from food, water, and the environment, as well as other illnesses suspected to result from commercially served or produced food to the municipal public health authority. Physician reporting and direct contacts with patients are the main ways in which foodborne illnesses come to the attention of municipal public health authorities. These authorities, in turn, are encouraged, but not required, to report the results of their investigations to the National Food Administration on a standardized form.

### Overview of the Study

The municipality of Uppsala has 186,000 inhabitants and includes the city of Uppsala, which is a center for research and education with two universities and a university hospital. In preparation for our study, we enhanced surveillance for foodborne illness in the municipality by adding analytical capabilities and staff to the municipal public health office and by providing information to the general public and the medical staff through media reports and at meetings. This information encouraged them to contact municipal public health inspectors by calling a dedicated telephone number to report cases of diarrhea, vomiting, or both, if they suspected food as the source of illness, and the affected person was a resident of the Uppsala municipality. Two types of incidents were distinguished: a) an outbreak, an incident in which two or more persons experienced a similar illness after ingestion of a common food and epidemiologic analysis implicated food as the source of the illness; and b) a single case, an incident in which one person became ill, with food as the suspected cause.

Address for correspondence: Roland Lindqvist, National Food Administration, P.O. Box 622, SE-751 26 Uppsala, Sweden; fax: 46-18-10-5848; e-mail: roli@slv.se

### Investigation of Outbreaks and Single Cases

Ill persons were interviewed by using a standardized questionnaire designed for this study (12); it contained questions about personal details (age, health, income), illness (symptoms, duration), animal contacts, and eating habits (what, where, with whom) the week before the illness.

When possible, food and fecal samples were collected and the implicated premises were inspected. Because resources were limited, criteria were developed for when to collect fecal samples. The goal was to collect feces from at least one ill person per incident, single or outbreak-associated. Samples were not collected if >2 days had passed since the diarrhea ended or if the person had been treated with antibiotics the week before sampling. Microbiologic evidence was not necessarily available from multiple cases to determine etiology in outbreaks. Fecal samples were analyzed for the following bacteria: *Aeromonas* spp., *Plesiomonas* spp., *Campylobacter* spp., *Salmonella* spp., *Shigella* spp., and *Yersinia enterocolitica*. Polymerase chain reaction (PCR) analysis (13) was performed for enterohemorrhagic *Escherichia coli*, enterotoxigenic *E. coli*, enteropathogenic *E. coli*, and enteroinvasive *E. coli*. The following viruses were analyzed: caliciviruses (PCR method [14], and electron microscopy [15]), rotaviruses, and astroviruses (electron microscopy [15]). In a few incidents, samples were analyized for *Vibrio* spp.

Relevant microbiologic analyses were performed on food remains if available. In their absence, other food samples were analyzed only if they could be presumed to have the same microbiologic properties as the suspected food.

Recorded incidents were classified as verified, probable, or possible foodborne incidents based on the following criteria. An incident was considered verified if the agent was isolated from both an ill person and the food. If the agent was isolated from either the food or an ill person, the incident was considered verified if a link between them could be determined on the basis of the information in the questionnaire but probable if the available information linking them was inconclusive. An outbreak could be classified as a probable incident entirely on the basis of information collected during the interview if the association was strong enough, e.g., several cases with similar symptoms and with only one meal in common. The remaining incidents that did not meet the exclusion criteria were considered possible foodborne incidents. An incident was excluded from the study if the ill person had traveled outside the country less than 1 week before onset of disease, if the investigation showed that the illness was not foodborne, or if the responses given during the interview of the affected person were insufficient. In total, 28 incidents were excluded.

### Retrospective Interview Study

To estimate the degree of reporting and incidence of foodborne illnesses in Uppsala, a retrospective interview study was performed 2 months after the study had ended. By random selection, 400 names were chosen from the telephone book. Persons answering the calls were interviewed by using a separate standardized questionnaire (12). If the person answering the phone was <15 years old, the interviewer asked to speak with an adult. In total, 266 persons were interviewed.

### Results

From February 1998 to January 1999, 268 incidents were recorded, compared with 31 to 44 incidents reported yearly between 1993 and 1997. Of the 268 incidents, 183 (68%) were single cases and 85 (32%) were outbreaks (Table 1). Collectively, 515 cases of foodborne illness were documented; interviews were conducted for 354 of these; 61% were women. Each week during the study period, 1 to 21 incidents were reported, but no obvious trend over the year was apparent (data not shown).

For 101 (38%) of the 268 incidents, 123 fecal samples were collected. A microbiologic agent was detected in 47 (38%) of the 123 fecal samples and in 45 (45%) of the 101 sampled incidents (Table 2).

In most incidents, no relevant food samples were available for analysis. In about one third of the 66 incidents in which food was sampled, potentially pathogenic microorganisms such as *Bacillus cereus*, *E. coli,* and *Staphylococcus aureus* were detected at levels between 1.3-5.9 log CFU $g^{-1}$ (Table 2).

In 213 (79%) of the 268 incidents, the etiologic agent was unknown because of insufficient information (Table 2). These incidents involved 334 (65%) of the 515 documented, single and outbreak-associated, cases. Bacteria caused 26 (10%) of the 268 incidents and 128 (25%) of the 515 documented cases; viruses caused 24 (9%) of the 268 incidents and 45 (9%) of the 515 documented cases (Table 2). The most common etiologic agent was calicivirus (20 incidents, 41 cases), followed by *Campylobacter* spp. (12 incidents, 16 cases) and *S. aureus* (5 incidents, 99 cases, Table 2).

Of the 268 incidents, 76 (8 single cases and 68 outbreaks) were classified as verified or probable (Table 3). These incidents resulted from ingestion of food prepared in a) restaurants or similar establishments (46%); b) homes (8%); c) grocery stores (4%); d) other places (2%); and e) unknown places (40%). The source of contamination was unknown in 27 incidents involving 58 ill persons. In 10 incidents (25 ill persons), only the meal, not the specific food item, was implicated. Meat and meat products (13 incidents, 34 ill persons) and mixed dishes (12 incidents, 128 ill persons) were the two most implicated food categories; poultry (5 incidents, 10 ill persons), beef (4 incidents, 7 ill persons), and sandwiches (4 incidents, 112 ill persons) were the most implicated subcategories.

One or more contributing factors could be identified in 18 (24%) of the 76 verified and probable incidents. These factors were a) lack of hygiene in processing, preparing, storing, and

Table 1. Number of single and outbreak-associated cases in incidents of foodborne illness detected in Uppsala municipality, Sweden, February 1998 through January 1999

| No. of cases in each incident | No. of incidents of indicated size (% of total incidents) | Total no. of cases in incidents of indicated size |
|---|---|---|
| 1 | 183 (68) | 183 |
| 2 | 59 (22) | 118 |
| 3 | 11 (4) | 33 |
| 4 | 5 (2) | 20 |
| 5 | 3 (1) | 15 |
| 6 | 2 (<1) | 12 |
| 7 | 2 (<1) | 14 |
| 13 | 1 (<1) | 13 |
| 14 | 1 (<1) | 14 |
| 93 | 1 (<1) | 93 |
| Total | 268 | 515 |

Table 2. Disease agents detected in feces and food samples and implicated[a] as etiologic agents in the investigated illnesses

| Agents | Detected in (no. of samples/incidents) | | Implicated in (no.) | |
|---|---|---|---|---|
| | Feces | Food | Incidents | Illnesses |
| Bacteria | | | | |
| *Bacillus cereus* | na[b] | 12/9 | 3 | 5 |
| *Campylobacter* spp. | 12/12 | 0 | 12 | 16 |
| EHEC[b] | 4/4[c] | 5/5[d] | 3 | 4 |
| EIEC[b] | 1/1 | na | 1 | 1 |
| EPEC[b] | 1/1 | na | 1 | 2 |
| ETEC[b] | 1/1[c] | na | 0 | 0 |
| *Salmonella* spp.[e] | 1/1 | 0 | 1 | 1 |
| *Staphylococcus aureus* | na | 10/9 | 5 | 99[f] |
| Total | 20/20 | 25/21[c] | 26 | 128 |
| Viruses | | | | |
| Astroviruses | 2/2 | na | 2 | 2 |
| Caliciviruses | 25/23[c] | na | 20 | 41 |
| Rotaviruses | 3/3[c] | na | 2 | 2 |
| Total | 29/27[c] | | 24 | 45 |
| Histamine | na | na | 2 | 3 |
| Several agents | [c] | | 3 | 5 |
| Unknown | | | 213 | 334 |
| Negative | 76/56 | 133/45 | | |
| Total agents | 123/101[g] | 158/66 | 268 | 515 |

[a]The agent was implicated as a cause of an illness incident on the basis of laboratory evidence, the interview, and assuming foodborne transmission.
[b]na = not analyzed; EHEC = enterohemorrhagic *Escherichia coli*; EIEC = enteroinvasive *E. coli*; EPEC = enteropathogenic *E. coli*; ETEC = enterotoxigenic *E. coli*.
[c]In two of the incidents, two (caliciviruses and EHEC) and three agents (calicivirus, ETEC, and rotaviruses), respectively, were detected in feces samples, and in two other incidents, two agents (*E. coli* and *B. cereus*, and *S. aureus* and *B. cereus*, respectively) were detected in food samples.
[d]Refers to generic *E. coli*. No further characterization was done.
[e]*Salmonella* Enteritidis (phage type 21).
[f]In the largest incident (93 cases), disease agents other than *S. aureus* may have been involved since atypically long incubation times were recorded for some of the cases.
[g]Sum minus negative does not equal the number of positive samples since two or more agents were detected in some samples. See footnote c.

Table 3. Causes of verified, probable, and possible foodborne incidents (single cases and outbreaks)

| Etiologic agent | No. single cases | No. outbreaks[a] (no. cases) | Total no. of single and outbreak-associated cases, by agent |
|---|---|---|---|
| Verified | | | |
| *Bacillus cereus* | 1 | 1 (3) | 4 |
| Calicivirus | 0 | 5 (23) | 23 |
| *Campylobacter* spp. | 0 | 2 (6) | 6 |
| Histamine | 1 | 1 (2) | 3 |
| *Staphylococcus aureus* | 1 | 3 (97) | 98 |
| Multiple agents[b] | 0 | 1 (2) | 2 |
| Subtotal | 3 | 13 (133) | 136 |
| Probable | | | |
| *B. cereus* | 1 | 0 (0) | 1 |
| Calicivirus | 0 | 2 (4) | 4 |
| *Campylobacter* spp. | 3 | 0 (0) | 3 |
| EHEC[c] | 0 | 1 (2) | 2 |
| EPEC[c] | 0 | 1 (2) | 2 |
| *S. aureus* | 1 | 0 (0) | 1 |
| Unknown | 0 | 51 (148) | 148 |
| Subtotal | 5 | 55 (156) | 161 |
| Possible | | | |
| Astroviruses | 2 | 0 (0) | 2 |
| Caliciviruses | 12 | 1 (2) | 14 |
| *Campylobacter* spp. | 7 | 0 (0) | 7 |
| EHEC | 2 | 0 (0) | 2 |
| EIEC[c] | 1 | 0 (0) | 1 |
| Rotavirus | 2 | 0 (0) | 2 |
| *Salmonella* Enteritidis | 1 | 0 (0) | 1 |
| Multiple agents[d] | 1 | 1 (2) | 3 |
| Unknown | 147 | 15 (39) | 186 |
| Subtotal | 175 | 17 (43) | 218 |
| Total | 183 | 85 (332) | 515 |

[a]An incident in which two or more persons experienced a similar illness after ingestion of a common food, and epidemiologic analysis implicated food as the source of the illness.
[b]An outbreak in which caliciviruses were detected in feces samples, and high levels of *B. cereus* and *S. aureus* were detected in suspected food samples.
[c]EHEC = enterohemorrhagic *Escherichia coli*; EPEC = enteropathogenic *E. coli*; EIEC = enteroinvasive *E. coli*.
[d]One incident in which rotaviruses, caliciviruses, and EHEC were detected in the feces sample from a single case, and one outbreak in which EHEC and caliciviruses were detected in the same feces sample.

handling food (11 incidents); b) temperature errors, i.e., inadequate refrigeration, cooking, or cooling (8 incidents); c) contamination by an infected person or equipment (5 incidents); d) cross-contamination from other products, ingredients, or the environment (4 incidents); e) contaminated raw food (2); and f) other factors (6).

Most (85%) of the 268 incidents were detected by a telephone call from an affected person, 13% (36/268) were detected through medical authorities, and 2% (5/268) were detected through other sources. However, in several incidents the caller had been in previous contact with the medical authorities.

The illness forced 122 (79%) of the 154 employed or self-employed patients to miss work (Table 4). Most patients never contacted medical authorities. In total, 45 (14%) of the 312 respondents included in this analysis had visited a doctor and 19 (6%) were hospitalized (Table 4). The average cost for a case of foodborne illness was estimated to be 2,164 Swedish Krona (SEK) ($246), of which 1,027 SEK ($116) were direct costs including doctor visits, hospitalization, and medicine; the rest were indirect costs (i.e., loss of production) (Table 4). The average expenses to a patient were estimated at 500 SEK ($57), mostly from loss of income. For a hospitalized patient, the average cost was 18,652 SEK ($2,117), time spent in the hospital was 3.1 days, and loss of production was 5.6 days.

Of the 266 respondents in the retrospective interview study, 10 persons (3.8%) suspected they had had a foodborne illness during the study period. This translates to an incidence of 38 illnesses per 1,000 inhabitants per year. Only 1 of the 10 affected respondents (10%) had called the municipal authority. Based on this degree of reporting, the actual number of foodborne incidents was estimated to be 2,700 (268/0.1) When the average number of illnesses per incident (1.9) was used, the number of illnesses per year was calculated to be 5,100 (1.9 x 2,700). This translates to an incidence of 28 illnesses per 1,000 inhabitants per year (5,100 illnesses / 186,000 inhabitants), which is in the same range as the first estimate.

### Discussion

Enhanced surveillance in combination with a telephone interview study was used to improve our understanding of foodborne illnesses and to address three limitations

Table 4. Estimated costs per case of foodborne illness

| Costs included | No. of persons[a] | Average[b] no. of visits or days | Average cost per illness[b] SEK[c] ($) | Min. SEK | Max. SEK |
|---|---|---|---|---|---|
| Direct costs | | | | | |
| Doctor visits | 45 | 0.2 visits | 173 | 0 | 3,714 |
| Hospitalization | 19 | 0.2 days | 809 | 0 | 43,150 |
| Medicine | 21 | na[d] | 5.3 | 0 | 200 |
| Other costs | 23 | na[d] | 40 | 0 | 4,200 |
| Total direct costs | 76[a] | | 1,027 (117) | 0 | 43,265 |
| Indirect costs | | | | | |
| Loss of production | 122 | 1.3 days | 1,137 | 0 | 17,934 |
| Total | 157[a] | | 2,164 (246) | 0 | 55,221 |

[a]Number of persons who reported a cost for each of the items in the questionnaire. Several persons reported more than one direct costs.
[b]Averages based on 312 persons answering the questions in the standardized questionnaire.
[c]$1 = 8.81 Swedish Krona (SEK) (May 2, 2000).
[d]na = not applicable.

commonly named in foodborne research (16): 1) underreporting; 2) lack of data on the incidence and severity of foodborne illnesses; and 3) lack of medical cost data on foodborne illness episodes, including those for which no medical care is sought. Surveillance was enhanced by improving some of its preconceived weaknesses, i.e., the awareness of foodborne illnesses and motivation to report them on the part of consumers and physicians and surveillance activities of the health authorities (8). This approach has both strengths and weaknesses. The advantages include the theoretical size of the study population (186,000 people), which makes the detection of rare disease agents possible, and the detection and investigation of single (17) and milder cases (3), not only outbreak-associated cases or cases occurring in persons who seek medical attention. Study limitations include the difficulty of defining the actual size and composition of the study population and establishing a case definition, which is partly based on suspicion. These limitations were addressed by conducting the retrospective telephone interview study and by classifying incidents on the basis of available evidence (Table 3).

Public attention raised by the study could have led to a shift from the normal underreporting to overreporting. However, the telephone interview study indicated that a bias towards underreporting still existed. Further, the use of this degree of reporting and the number of illnesses per incident yielded an annual incidence estimate that was in reasonable agreement with the first estimate based on the telephone interview study (28 and 38 illnesses, respectively, per 1000 inhabitants). The uncertainty of the second estimate is probably greater than the first since it is based on only 10 suspected cases, and the average number of illnesses per incident is probably an underestimate because of the large proportion of single cases.

The annual incidence estimates obtained from our telephone interview study and from a national interview study (11) were also in reasonable agreement, 38 compared to 79 illnesses per 1,000 inhabitants. In comparison, 6.5 to 33 million cases of food-related illness per year have been estimated to occur each year in the United States (18). This translates to an incidence (25 to 130 cases per 1,000 inhabitants) similar to that in our study. A more recent report estimated the annual U.S. incidence of foodborne illnesses to be 278 cases per 1,000 inhabitants (4). However, caution should be exercised when comparing incidence estimates from different studies since they may partly reflect differences in the surveillance systems used and the assumptions behind the estimations.

Based on the number of foodborne illnesses reported after this study (130 and 100 incidents reported in 1999 and 2000, respectively), improved detection appears to be persisting. The average annual number of incidents in 1993-97 was 40, which (by using the estimated actual number of incidents, 2,700) indicates underreporting by a factor of 67 (2,700/40).

Based on data from 1987 (19), the cost per case of salmonellosis in Sweden can be estimated at $1,322 (converted from United Kingdom Pounds), which is much higher than our estimate for the cost per case of foodborne illness (Table 4). Our lower estimate is not unexpected since it is based on illnesses caused by a variety of agents and a spectrum of symptoms, from mild to more severe. Comparing costs between countries is difficult since the methods, types of illnesses, and health-care systems may differ. Razem and Katusin-Razem (20) estimated the cost per case of salmonellosis in Croatia to be $284 by adjusting estimates from different countries based on the ratio of their gross national products. In New Zealand (21), the estimated cost per case of foodborne infectious disease, $200, was in the same range as our estimate, whereas a considerably higher cost, $1,250, was estimated for a case of foodborne illness in the United States (22). The New Zealand estimate, however, was based on infectious diseases only, and the second estimate included costs for business losses, deaths, legal settlements, and investigation (22). Our estimate did not include the latter costs nor costs resulting from potential medical sequelae (5,6) and personal consequences not usually estimated in monetary terms (3). By combining the present cost per illness with the previously estimated 500,000 cases of foodborne illness per year in Sweden (11), the costs to society can be estimated at 1,082 million SEK ($123 million).

Both the present data and those from voluntary reports from the local authorities to the National Food Administration (10) indicate that a substantial proportion of foodborne illnesses occur because of mistakes in or a lack of knowledge of food-handling procedures at commercial food establishments. Another similarity is the relatively large proportion of incidents with unknown causes. These comparisons indicate that the surveillance system gives useful information but also has several limitations (2). A lower proportion of incidents in which *Salmonella* spp. was implicated was found in this study (Table 2), compared to other reports of foodborne illnesses both in North America (8) and in Europe (23). It is not likely that this result is due to a sampling bias since fecal samples were analyzed for salmonellae in the same frequency as for caliciviruses and *Campylobacter* spp. Instead, it may reflect the low prevalence in Sweden of salmonella in food, cattle, pigs, and poultry (<1%) because of an extensive control program (10).

The study failed to establish a rapid link between the physicians and the municipal public health inspectors as indicated by a review of communicable diseases reported to the CMO during the study period. Indeed, at least 27 incidents, which according to the public health inspectors were possible foodborne illnesses, were not reported. These incidents were not part of the study. This means that serious incidents may go undetected and that valuable time is lost since the CMO generally receives notification more than a week after the incident occurs. Also, the number of incidents forwarded from medical staff in the different areas in the municipality varied. This may reflect a true difference in incidence but more likely is a reflection of their motivation to participate in the study. Guzewich et al. (9) stressed the importance of the motivation of those involved in the surveillance and suggested improved feedback to stimulate motivation.

On the basis of the results and experiences obtained during our study (12), several suggestions to improve detection of foodborne illness incidents can be proposed. First, information should be directed to the general public and the medical staff to motivate them to report suspected incidents to the local public health authorities. To meet the increased number of reported incidents, probably mostly involving persons not in need of medical care, and to optimize use of available resources, the municipal public health authorities should develop criteria for when and how to investigate incidents. The minimum requirement should be conducting an interview of the affected person according to a standardized questionnaire. Detection and investigation of incidents can be improved by arranging opportunities for medical and public health staff to meet on a regular basis and by establishing channels for rapid communication. This will facilitate both cooperation and coordination. These groups also need more training in food safety and in modern techniques to investigate incidents. Reporting of investigated incidents to the National Food Administration should be facilitated through a web-based system, and the feedback to those contributing should be improved. Foodhandlers in restaurants and similar establishments should be educated regarding food hygiene. Finally, to improve our knowledge, the existing passive surveillance should be supplemented with additional studies and approaches.

### Acknowledgments

We are grateful to the numerous people contributing to this study, including medical and laboratory staff, the project steering group, and all the study participants in Uppsala who patiently answered the questionnaire and provided samples for analysis.

Dr. Lindqvist is a microbiologist in the department of research and development at the National Food Administration in Sweden. His research interests include the epidemiology of foodborne illness, risk assessment of foodborne hazards, and PCR detection methods for bacterial pathogens in food.

### References

1. Käferstein FK. Food safety: a commonly underestimated public health issue—Introduction. World Health Statistics Quarterly 1997;50:3-4.
2. Notermans S, Borgdorff MW. A global perspective of foodborne disease. J Food Prot 1997;60:1395-9.
3. Buzby JC, Roberts T. Economic costs and trade impacts of microbial foodborne illness. World Health Statistics Quarterly 1997;50:57-66.
4. Mead PS, Slutsker L, Dietz V, McCaig LF, Bresee JS, Shapiro C, et al. Food-related illness and death in the United States. Emerg Infect Dis 1999;5:607-25.
5. Smith JL. Arthritis and foodborne bacteria. J Food Prot 1994;57:935-41.
6. Lindsay JA. Chronic sequelae of foodborne disease. Emerg Infect Dis 1997;3:443-52.
7. Motarjemi Y, Käferstein F. Global estimation of foodborne diseases. World Health Statistics Quarterly 1997;50:5-11.
8. Bean NH, Goulding JS, Daniels MT, Angulo FJ. Surveillance for foodborne disease outbreaks—United States, 1988-1992. J Food Prot 1997;60:1265-86.
9. Guzewich JJ, Bryan FL, Todd ECD. Surveillance of foodborne disease I. Purposes and types of surveillance systems and networks. J Food Prot 1997;60:555-66.
10. Lindqvist R, Andersson Y, de Jong B, Norberg P. A summary of reported foodborne disease incidents in Sweden, 1992 to 1997. J Food Prot 2000;63:1315-20.
11. Norling B. Food poisoning in Sweden—results of a field survey. Report No. 41/94. Uppsala, Sweden: National Food Administration; 1994.
12. MAT UPP--intensivstudie av matförgiftningar i Uppsala kommun under ett år. Livsmedelsverket. SLV rapport 12/99. (In Swedish).
13. Svenungsson B, Lagergren Å, Ekvall E, Hedlund K-O, Kärnell A, Löfdahl S, et al. Enteropathogens in adults with diarrhea and healthy controls: a one-year prospective study in a Swedish clinic for infectious disease. Clin Infect Dis 2000;30:770-8.
14. Qiao H, Nilsson M, Rubilar Abreu E, Hedlund K-O, Johansen K, Zaori G, et al. Viral diarrhea in children in Beijing, China. J Med Virol 1999;57:390-6.
15. Hedlund K-O, Bennet R, Eriksson M, Ehrnst A. Norwalk-like virus as a cause of diarrhea in a pediatric hospital. Clinical Microbiology and Infection 1998;4:417-21.
16. Buzby JC. Data on foodborne disease cases, severity and costs. In: Roberts T, Jensen H, Unnevehr L, editors. Tracking foodborne pathogens from farm to table: data needs to evaluate control options. Miscellaneous pub. no. 1532. Washington: U.S. Department of Agriculture, Economic Research Service, Food and Consumer Economics Division; 1995. p. 3-30.
17. Tauxe RV. Emerging foodborne diseases: an evolving public health challenge. Emerg Infect Dis 1997;3:425-34.
18. Foodborne pathogens: risk and consequences. Ames (IA): Council of Agricultural Science and Technology; 1994.
19. Persson U, Jendteg S. The economic impact of poultry-borne salmonellosis: how much should be spent on prophylaxis? Int J Food Microbiol 1992;15:207-13.
20. Razem D, Katusin-Razem B. The incidence and costs of foodborne diseases in Croatia. J Food Prot 1994;57:746-53.
21. Scott WG, Scott HM, Lake RJ, Baker MG. Economic cost to New Zealand of foodborne infectious disease. N Z Med J 2000;113:281-4.
22. Todd ECD. Cost of acute bacterial foodborne disease in Canada and the United States. Int J Food Microbiol 1989;9:313-26.
23. Simone E, Goosen M, Notermans S, Borgdorff MW. Investigations of foodborne diseases by food inspection services in the Netherlands, 1991 to 1994. J Food Prot 1997;60:442-6.
